# Propionic Acid Groups and Multiple Aromatic Rings Induce Binding of Ketoprofen and Naproxen to the Hydrophobic Core of Bovine Serum Albumin

**DOI:** 10.1021/acs.molpharmaceut.3c00169

**Published:** 2023-06-20

**Authors:** Minori Tsurushima, Yuya Kurosawa, Satoru Goto

**Affiliations:** Faculty of Pharmaceutical Sciences, Division of Colloid and Surface Science, Research Institute for Science and Technology, Tokyo University of Science, 2641 Yamasaki, Noda, Chiba 278-8510, Japan

**Keywords:** serum albumin, singular value decomposition, fluorescence, drug adsorption, hapten

## Abstract

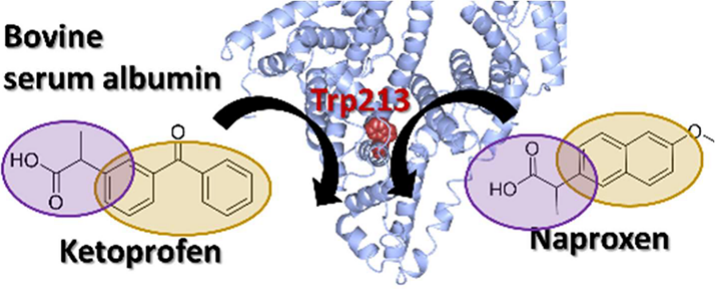

Ketoprofen (KP), which causes photosensitivity by interacting with serum albumin (SA), and three drugs, ibuprofen (IBP), naproxen (NPX), and diazepam (DZP), which share the same binding site, were investigated for their interaction with bovine SA (BSA). For KP, DZP, and IBP, where drug-concentration-dependent quenching of BSA-intrinsic fluorescence was observed, a modified Stern–Volmer plot showed that dynamic quenching was dominant for KP and static quenching was dominant for DZP and IBP. However, this alone cannot be compared with NPX. Therefore, by performing singular value decomposition (SVD) fluorescence spectroscopy, we were able to find the behavior of the drug-concentration-dependent Langmuir-type principal component vectors. *K*_SVD_ obtained by the Langmuir equation showed a high correlation with the static extinction constant *V*. Therefore, *K*_SVD_ indicates the association constant of the drug with BSA and it was found that NPX and IBP had higher values than KP. Finally, in the analysis of the temperature factors of amino acid residues in each drug-binding region and Trp residues, KP and NPX significantly reduced these temperature factors whereas DZP and IBP hardly changed them. This result is consistent with the dynamic and static quenching dominance in the total quenching mechanism. Summarizing the results so far, it was shown that penetration into the hydrophobic core inside BSA can be achieved not only by one of the multiple aromatic rings and propionic acid groups but also by the joint effect of both. In this study, SVD enabled us to extract information on drug adsorption to BSA from fluorescence spectra. Furthermore, the application of SVD is expected to make it possible to perform fluorescence analysis for drug binding to proteins without being limited by the fluorescence properties of the drug.

## 1 Introduction

Serum albumin (SA) is the most abundant protein in plasma and is a carrier for drug transport in the body.^1−3^ Two major drug-binding sites are known as Sudlow sites, which are classified as warfarin site I and benzodiazepine site II.^4^ They serve as carriers for a wide range of drugs, from NSAIDs to anticancer drugs.^4^ In addition to the transport of drugs administered into the body, SAs are also attracting attention in the development of drug delivery systems such as albumin nanoparticles.^1^ However, ketoprofen (KP) is known to bind to SA and cause SA haptenization as a cause of drug-induced photosensitivity.^5^ Thus, it is clear that drug binding and associated conformational changes in SA can lead to serious side effects. Of course, this is also the case for the SA-based drugs discussed above. Therefore, the classification of drug structure and binding mode to SA is an important research issue for the development of safe drugs now and in the future. There are similar reports not only for NSAIDs such as KP but also for UV absorbers. This includes oxybenzone and avobenzone, which have a benzophenone skeleton like KP.^6^ Analysis of binding modes of organic compounds to albumin is important not only in the medical field but also in everyday life such as cosmetics. In this study, we focused on site II of SA to which KP, which is known to cause drug-induced photosensitivity, binds. We attempted to classify the binding mode of KP and another site II drugs to SA by fluorescence analysis using the Stern–Volmer plot^7^ and singular value decomposition (SVD).^8^ The results of this study are summarized as follows. Bovine serum albumin (BSA) was used as the representative SA. The model drugs used were all site II binding drugs, KP, ibuprofen (IBP), naproxen (NPX), and diazepam (DZP).

The Stern–Volmer equation is a fluorescence analysis widely used to evaluate protein-drug binding.^7^ The fluorescence of aromatic amino acids such as tryptophan, phenylalanine, and tyrosine in proteins^9^ is quenched by the energy transfer associated with drug binding. The drug concentration-dependent quenching can be evaluated using a Stern–Volmer equation to calculate the quenching constant that indicates the affinity of the drug for the protein.^10,11^ However, the conventional Stern–Volmer equation has two types of quenching, static quenching due to drug binding and dynamic quenching due to collision,^7,12^ and it is necessary to focus only on static quenching to discuss the affinity of drugs and proteins. Currently, this is achieved by an analysis called a modified Stern–Volmer equation (Lehrer equation).^7,12^ This study also mainly uses the modified Stern–Volmer equation. However, it is only an analysis method for quenching and cannot be applied to drugs that enhance fluorescence with the addition of drugs.

SVD was used in this study to evaluate drugs that increase or quench fluorescence with the addition of drugs on the same scale. SVD is a multivariate analysis used for dimensionality reduction and has been used for UV–vis,^13^ electron spin resonance,^14^ Fourier transform infrared spectroscopy (FTIR),^15^ circular dichroism (CD),^16^ and fluorescence spectra.^17^ Fluorescence and CD spectra, in particular, are used in studies of protein conformation, as in this study. SVD processing can extract the dominant components of the data set of interest that are increasing or decreasing in a concentration- or time-dependent manner. What is important is the meaning of each extracted component. For example, in the analysis of CD spectra, the two major components are the amount of α-helix and β-sheet, respectively, which were elucidated by intentionally making the proteins α-helix-rich using trifluoroethanol and by including α-helix-rich and β-sheet-rich proteins in the data set. In the fluorescence spectra, the peak shift occurs in a protein structure-dependent manner.^18^ Since SVD is linearly coupled, it is difficult to analyze data sets in which the basis spectra vary in this way. However, by adding spectral data created by Gaussian functions to the data set, as in supervised learning in machine learning, it was possible to interpret the meaning of each component.^17^ This method also plays an important role in SVD, which is the subject of this study. Albumin is a carrier for a variety of drugs and is of interest in DDS research, but the immunogenicity associated with drug binding can cause potentially fatal side effects. In this study, we investigated the mechanism by which drugs causing photosensitivity bind to BSA, with the aim of furthering research on immunogenicity resulting from drug–protein interactions.

In this study, modified Stern–Volmer plots and SVD binding patterns to BSA site II showed that NPX belonged to the same group as KP. Since NPX has a greater affinity for BSA than KP, it may be easier to haptenize BSA than KP. Classification of the binding mode of each drug by these methods leads to the prevention of potential drug-induced allergies including photosensitivity. In the future, this method can be used to optimize the composition of not only small-molecule drugs but also emulsions such as vaccines, nonionic surfactants such as lipid nanoparticles, and drugs containing polymers such as phospholipids.

## 2 Experimental Section

### 2.1 Materials

BSA (product code 017-21273), KP (116-01092), DZP (045-18901), and NPX (147-07201) were purchased from FUJIFILM Wako Pure Chemical Industries (Osaka, Japan). IBP (I0415) was purchased from Tokyo Chemical Industries (Tokyo, Japan). The purity of BSA is 95.0% or higher and 98.0% or higher for four drugs. All other reagents were of the highest commercially available grade.

It should be noted that the concentration of each drug used does not perfectly replicate the clinical environment. Compared to in vivo, the BSA concentration in this experiment was 1/100 times higher and the KP concentration was 100 times higher. However, the clinical drug concentration does not mean that the drug does not bind to the BSA. The concentrations are the minimum necessary to obtain information on the binding mode of drugs and conformational changes of BSA by physicochemical methods.

### 2.2 Fluorescence Measurements

All fluorescence measurements were conducted using a Shimadzu RF-5300PC spectrofluorophotometer (Shimadzu, Kyoto, Japan). All fluorescence experiments, including sample preparation, were performed in an air conditioner at 25 °C. The excitation and emission slit widths were 1.5 and 3.0 nm, respectively. The fluorescence spectra were measured at an excitation wavelength of 284 nm for Trp. In the experiment, the solvent at pH 6.4 was 50 mM citrate buffer and the solvents at pH 7.4 and pH 8.4 were Tris–HCl buffer. The BSA concentration is 5.0 μM for all samples. At shorter wavelengths, specifically 278 and 262 nm, Tyr and Phe are also excited.^19^ It has also been shown that Tyr fluorescence overlaps with Trp fluorescence at the excitation wavelength of 275 nm.^20^ Furthermore, the excitation wavelength of NPX is located around 270 nm^21,22^ and it is clear that the fluorescence of NPX alone is too strong at shorter wavelengths. Therefore, in consideration of these factors, 284 nm was chosen as the excitation wavelength in this study.

### 2.3 Quenching Analysis of BSA Intrinsic Fluorescence by the Stern–Volmer Plot

The classical Stern–Volmer equation is expressed as follows.
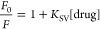
1

Here, *F*_0_ is the fluorescence intensity without a drug, *F* is the fluorescence intensity in the presence of each drug, and [drug] is the drug concentration.

The Stern–Volmer quenching constant *K*_SV_ has implications for both dynamic and static quenching. It is the modified Stern–Volmer equation shown in eq 2 that can be calculated separately.
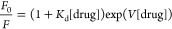
2

Here, *K*_d_ is the dynamic extinction constant and *V* is the static extinction constant.

In addition, the eq 3 related to the Stern–Volmer equation used for the purpose of calculating the association constant *K*_a_ and the number of binding sites *n* of a drug with a protein is shown below.^23^
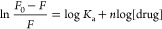
3

We evaluated the quenching of BSA intrinsic fluorescence using the above three equations.

### 2.4 Reducing Procedure of Dimensionality by SVD in Fluorescence Spectra and Interpretation Approaches for Imitation Spectra with a Set of Gaussian Functions

The *i*th observed fluorescence spectrum {Φ_*i*_→|1 ≤ *i* ≤ *n*} of the sample is represented as the *m*-dimensional vertical vector measured at the wavelength {λ_*j*_|1 ≤ *j* ≤ *m*}. The matrix M comprised a horizontal sequence of vectors from the first spectral vector through the *i*th spectral vectors, with an *m* × *n* rectangular matrix defined as follows, where *m ≥ n*:
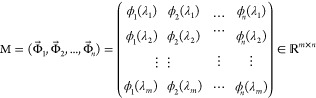
4

M and M^*t*^ are real and transposed matrices, respectively. Their products M^*t*^M and MM^*t*^ become orthogonal matrices, and eigenvectors are the rows of U and V. The matrices describing M are transformed into the formulas
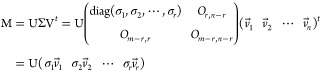
5

The diagonal matrix Σ constitutes the diagonal elements {σ_*i*_|1 ≤ *i* ≤ *r*} of the positive real values in descending order. These elements are singular values, indicating dispersion. The *i*th column of the orthogonal matrix V is the coefficient vector corresponding to the singular value σ_*i*_, and the vector *v⃗_i_* is called a singular vector. The principal component vector ω⃗_*i*_ was the coefficient vector *v⃗_i_* multiplied by the corresponding singular value σ_*i*_.
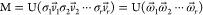
6

The matrix U has rows that are the basis function vectors. From the diagram for the logarithm of the singular value in descending order versus the index corresponding to the documental spectra, we practically decided the dimensionality, namely, the minimum dimensionality of the basis functions required to reproduce the vector space of the documental spectra. It might be practically negligible with singular values less than a several hundredth of the highest singular value of the first principal components. Due to the dimensionality *r* decided under this criterion instead of the mathematical rank ρ, the yielded principal components approximately reproduce the vector space including the documental spectrum as the *j*th feature vector *x_j_*→ composed of the *i*th elements *x*_*i*, *j*_:
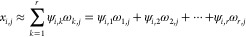
7

Figure S1 shows the model fluorescence spectra created by a Gaussian function (GFMs), in which only the intensity of the fluorescence peak changes, only the wavelength changes, or both change. The GFMs were subjected to SVD together with measured fluorescence spectra. This method is based on a previously reported content.^17^Equation 8, shows the Gaussian function. The behavior of fluorescence intensity was reproduced by *A* values, and the behavior of fluorescence wavelength was reproduced by *B* values. All GFMs were created by a single Gaussian function, and all *C* values are the same.
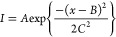
8

A schematic diagram is provided in Scheme S1 to facilitate understanding of the SVD process for fluorescence spectra.

### 2.5 Drug Adsorption Analysis by ω_3_ Obtained from SVD

Drug adsorption to BSA has been analyzed as Langmuir-type adsorption isotherm, and the present study follows this pattern. If ω_3_ is the response to drug adsorption, the following equation holds.
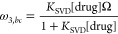
9

Here, ω_3,bc_ is baseline-corrected so that the starting point of ω_3_ is the origin. *K*_SVD_ is the association constant, [drug] is the concentration of each drug, and Ω is ω_3,bc_ at saturation adsorption. Curve fitting was performed on the measured values using eq 9.

### 2.6 Temperature Factor Analysis of Serum Albumin and Its Drug Complex Crystals

The crystal structures of BSA, BSA–KP complex, BSA–NPX complex, HSA, HSA–DZP complex, and HSA–IBP complex obtained from PDB were analyzed. The temperature factors of amino acid residues or tryptophan residues bound to each drug were calculated by CCP4i. The former used the average value of 6–14 amino acids. The relative temperature factor was calculated by dividing the temperature factor of the complex by the temperature factor of the SA alone.

## 3 Results and Discussion

### 3.1 Affinity to BSA of Drugs that Quench BSA-Intrinsic Fluorescence upon Binding

**Figure 1:**
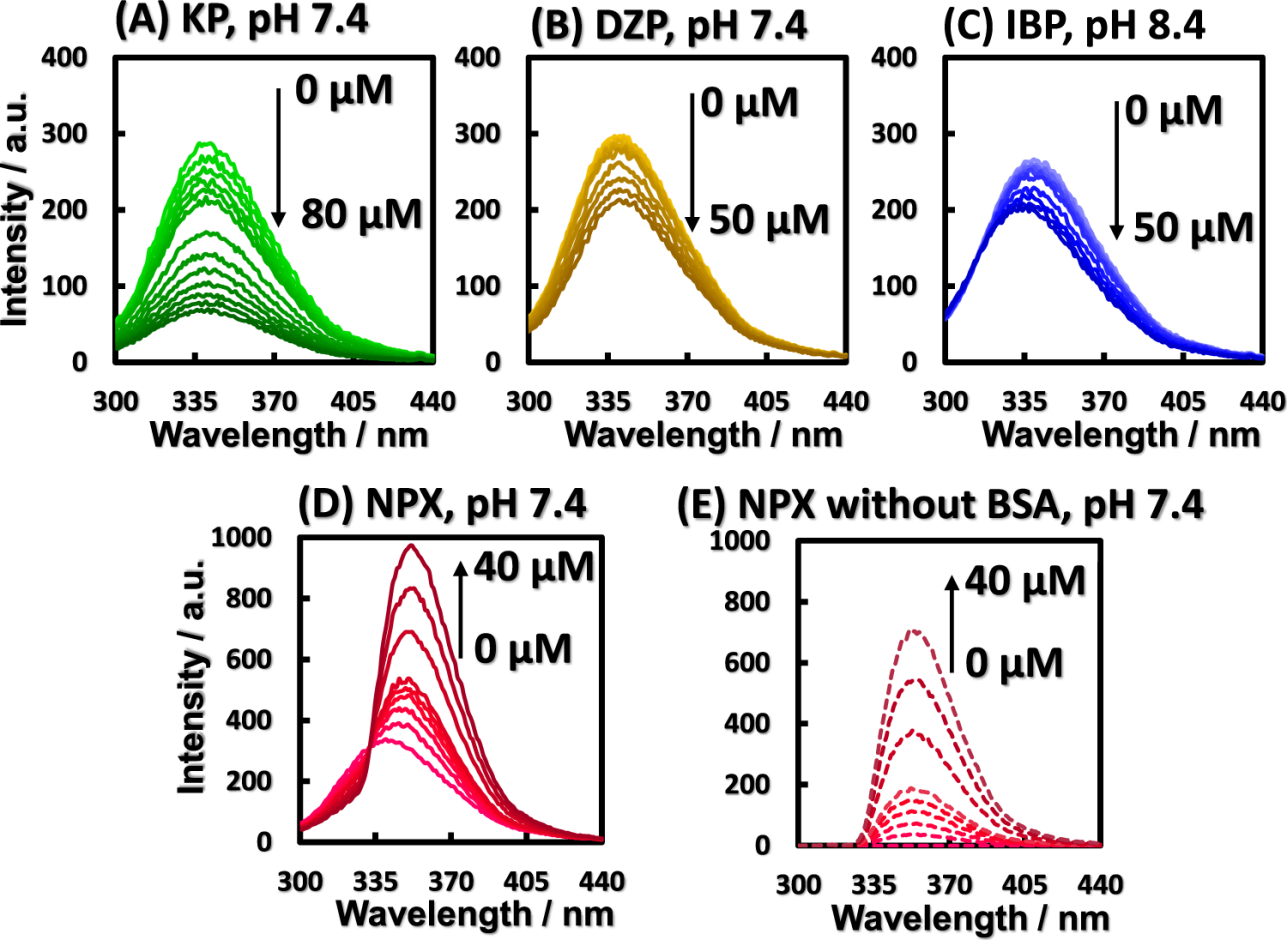
Drug-concentration-dependent quenching and increase of BSA intrinsic fluorescence. Fluorescence spectra of BSA with (A) KP at pH 7.4, (B) DZP at pH 7.4, (C) IBP at pH 8.4, (D) NPX at pH 7.4, and (E) NPX at pH 7.4 in the absence of BSA. [BSA] = 5.0 μM.

In order to distinguish the binding mode of each site II binding drug, the behavior of BSA intrinsic fluorescence upon drug binding was evaluated. Tyr and Phe absorb at shorter wavelengths than Trp, have weaker absorption, and are less sensitive to the surrounding environment. Furthermore, they transfer energy to nearby Trp. Therefore, the fluorescence of Trp is the main factor in the behavior of protein intrinsic fluorescence.^24^Figure 1 shows the concentration-dependent intrinsic fluorescence spectra of each drug. Measurements were taken at pH 6.4, 7.4, and 8.4, and Figure 1 shows the result at pH 7.4 or the fluorescence spectrum at the pH at which the characteristic result occurred. The rest of the measured spectra are shown in Figure S2. Figure 1A and B show that KP and DZP quenched the BSA intrinsic fluorescence without any peak shift, respectively. This indicates that the polarity around Trp residues did not change with the binding of KP and DZP.^11^ On the other hand, as shown in Figure 1C, IBP blue-shifted the intrinsic fluorescence of BSA with quenching, suggesting that IBP enhances the hydrophobic environment around Trp residues upon binding to BSA, i.e., burial in the hydrophobic core.^11^ In Figure 1D, NPX showed exceptional results compared to the other three drugs. A concentration-dependent red shift and increase in fluorescence intensity of NPX were observed. The reason is that NPX has autofluorescence, which is absent in KP, DZP, and IBP. The fluorescence spectra of NPX in the absence of BSA is shown in Figure 1E. When the fluorescence spectrum in the absence of BSA was subtracted from that in the presence of BSA as a general method of removing the baseline, the concentration dependence was broken and the BSA intrinsic fluorescence increased or decreased with the NPX concentration. This indicates that NPX may exchange fluorescence energy with Trp residues of BSA. This is explained in more detail below. The fluorescence of NPX alone was monotonically enhanced in a concentration-dependent manner. If Trp fluorescence is quenched in an NPX concentration-dependent manner, as is the case with other drugs, subtracting the fluorescence of NPX alone from the fluorescence spectrum of BSA in the presence of NPX should result in an NPX concentration-dependent quenching. However, the Trp fluorescence of BSA was actually increased at NPX concentrations up to 6.0 μM and decreased at higher concentrations (Figure S3). Therefore, we hypothesized that the presence of Trp increased or decreased the fluorescence of NPX.

**Figure 2:**
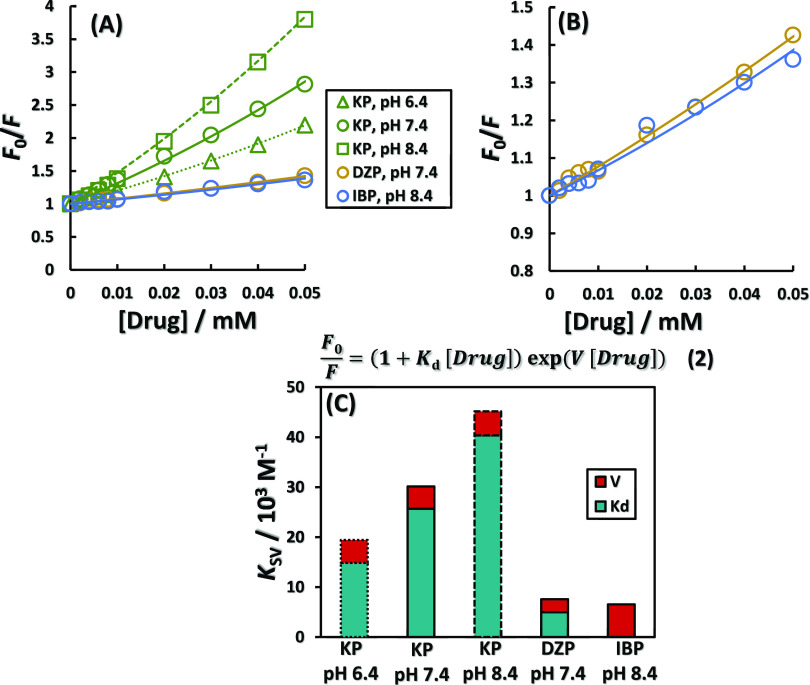
Application of modified Stern–Volmer plots (A) to drug concentration-dependent quenching of BSA intrinsic fluorescence. (B) Enlarged view of DZP and IBP plots and curves in Figure 2A. (C) Static quenching constant (*V*) and dynamic quenching constant (*K*_d_) for KP, DZP, and IBP.

As shown in Figure S2D and F, DZP showed no significant quenching at pH 6.4 and pH 8.4, respectively, i.e., no binding to BSA. As shown in Figure S2G and H, IBP showed no significant quenching, i.e., no binding to BSA, at pH 6.4 and pH 7.4, respectively. The increased binding of DZP and IBP above neutral pH was attributed to the NB transition of BSA.^25^ Similar results have also been reported.^26^ However, no quenching of DZP occurred at pH 8.4. This is because the disulfide bonding, which rapidly increases around pH 9,^27^ changes the distribution of sulfur atoms to which the chloro group of DZP can bind; the distance between Trp and DZP changes; and the binding of DZP is not reflected in the quenching of BSA intrinsic fluorescence. Here, we evaluated the quenching for KP, DZP, and IBP, to which the Stern–Volmer equation is applicable. Figure 2A shows the modified Stern–Volmer plot for each drug. Figure 2B shows the enlarged plots for DZP and IBP, which have smaller slopes, for better visibility. Figure S5 includes the results for higher concentrations of KP. The modified Stern–Volmer equation (eq 2) is an analysis that can be applied when static and dynamic extinctions occur simultaneously. The second-order Stern–Volmer equation also exists, but it has a symmetric relationship between the dynamic and static quenching constants. In addition, to use this equation, the dynamic quenching constant is usually assumed based on viscosity and temperature and the static quenching constant is calculated.^7^ Therefore, the modified Stern–Volmer equation was used here to separate static and dynamic extinction. The dynamic quenching constant *K*_d_ and static quenching constant *V* obtained from the analysis are shown in Figure 2C. For KP, only *K*_d_ increased in a pH-dependent manner while *V* remained almost unchanged. In contrast to KP, DZP and IBP had a large ratio of *V* to the total quenching constant *K*_SV_ (34%). The pH-dependent increase in *K*_d_ for KP can be attributed to the NB transition discussed earlier. The large percentage of *V* in DZP and IBP can be attributed to the lower selectivity of their binding regions compared to KP. The static quenching constant *V* is discussed as the effective volume and indicates the spatial extent of the area where the drug is present when quenching occurs.^28,29^ DZP and IBP are considered to exhibit non-selective binding due to their proximity to the disulfide bond and surfactant-like properties,^30^ respectively, resulting in a large *V*. The static quenching constant is a parameter corresponding to the association constant, indicating that KP clearly binds more readily to BSA than DZP and IBP. The results applying the classical Stern–Volmer equation are shown in Figure S4. Since this analysis does not distinguish between dynamic and static quenching,^29^ we focus here on the modified Stern–Volmer equation described above. The analysis using eq 3 to calculate the number of coupling sites and the association constant associated with the Stern–Volmer equation is shown in Figure S7. Figure S7A is the result of fitting eq 3 to the measured data. The obtained association constant *K*_a_ is shown in Figure S7B, and the number of binding sites *n* in Figure S7C. *K*_a_ shows the same trend as that of *K*_SV_ in Figure 2. The number of binding sites *n* is approximately 1 for KP, DZP, and IBP. The binding as a multimer was ruled out as the diversity of the binding mode.

Analysis of quenching cannot tell us anything about the binding of NPX to BSA, nor can it be compared to KP, DZP, or IBP, of course. Therefore, we performed SVD on all measured fluorescence spectra to evaluate the effects of these drugs, including NPX, on BSA intrinsic fluorescence on the same scale.

### 3.2 SVD Including GFMs Extracts Features that Are Difficult to Understand Visually in the Spectra

For drugs other than NPX, quenching analysis based mainly on the Stern–Volmer equation was applicable. In order to evaluate the binding to BSA, including NPX, all measured fluorescence spectra were subjected to SVD, including the GFM shown in Figure S1, and the fluorescence spectra in Figure 1 and Figure S2 were subjected to SVD in order to understand the meaning of each component obtained by the SVD process. This method is inspired by supervised learning in machine learning, and examples have already been reported.^17^ The details are shown in the Experimental Section.

**Figure 3:**
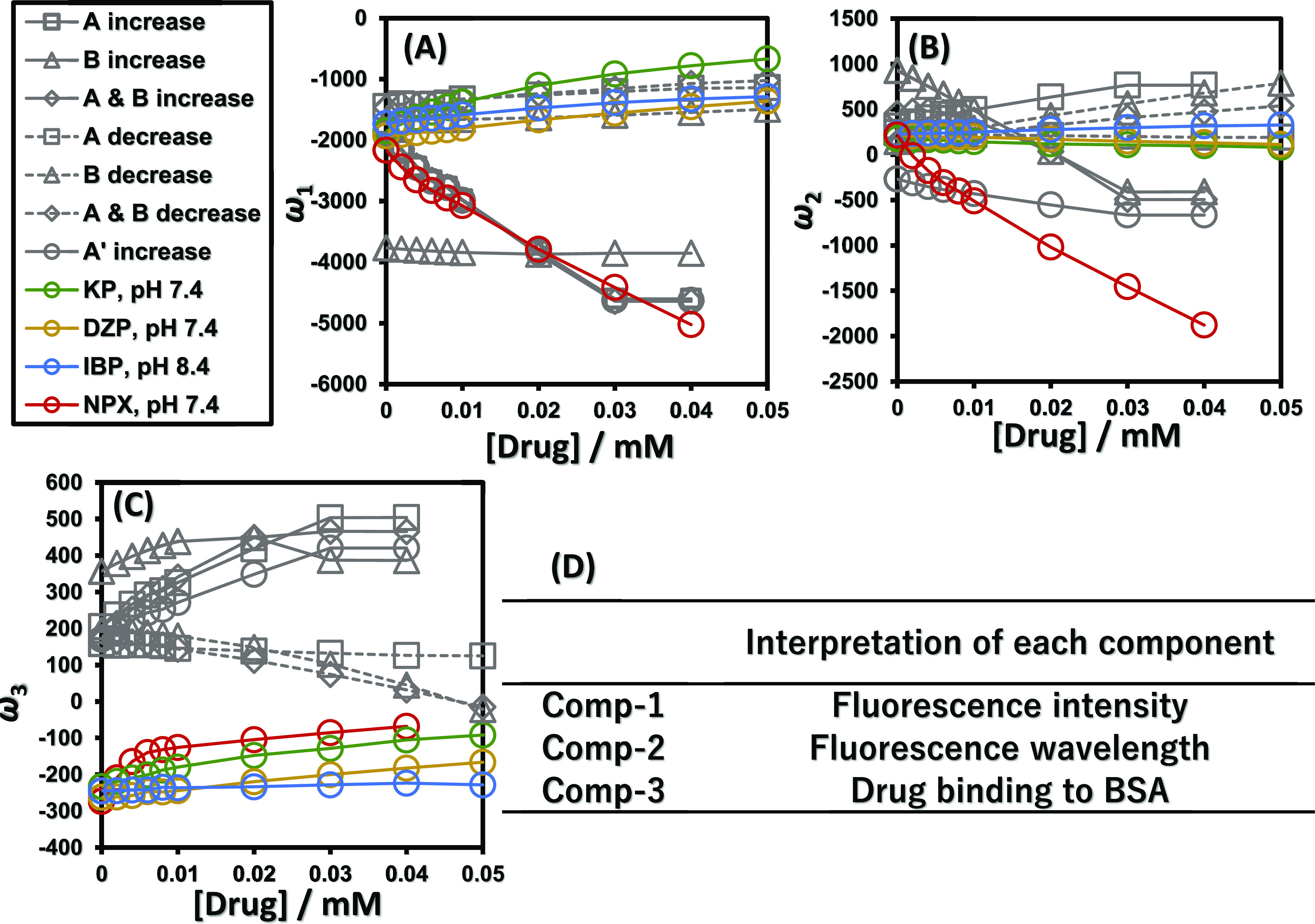
Interpretation of each component by SVD including GFM. Variation of ω_1_ (A), variation of ω_2_ (B), and variation of ω_3_ (C) with respect to drug concentration. Green, yellow, blue, and red lines are experimental values. Gray solid and dashed lines are the GFM value. (D) Interpretation of each SVD component obtained by GFM.

The resulting singular values of the SVD are shown in Figure S8A. We decided to focus on the first, second, and third components as the main component that can adequately reproduce the original data set. The basis vectors up to the third component are shown in Figure S8B. It is found that the original data set is represented by the increase or decrease of these three basis vectors. Figure 3 shows the principal component vector ω_*i*_, which is the product of the singular value σ_*i*_ and the singular vector λ_*i*_ for each component. Figure S8 shows the concentrations up to the denser concentrations for KP, but since they show a similar trend, we will proceed with the inference based on Figure 3. The interpretation of each component obtained by SVD is summarized in Scheme S2. Briefly, ω_1_ reflects a fluorescence intensity behavior, ω_2_ reflects a fluorescence wavelength behavior, and ω_3_ reflects drug binding to BSA. A detailed explanation is given below. In the first component, the principal component vectors ω_1_ of the GFMs *A* increase, *A*&*B* increase, and *A*′ increase, where the peak intensity, *A*, was increased, decreased uniformly. The same trend was observed for NPX, whose fluorescence intensity also increased in a concentration-dependent manner. The principal component vectors ω_1_ of *A* decrease and *A*&*B* decrease, and GFMs with *A* decreased, uniformly increased. The same trend was observed for KP, DZP, and IBP, which also showed a concentration-dependent decrease in fluorescence intensity. On the other hand, *B* increase and *B* decrease, in which only the peak wavelength, *B*, changed, showed no change. This indicates that the first component reflects the increase or decrease in fluorescence intensity in the fluorescence spectrum. In the second component, the principal component vectors ω_2_ of *B* increase and *A*&*B* increase, which are GFMs in which the peak wavelength, *B*, is increased, decreased uniformly. The same trend was observed for NPX, whose fluorescence wavelength increased in a concentration-dependent manner. The principal component vector ω_2_ of *B* decrease and *A*&*B* decrease, which are GFMs with *B* decreased, increased uniformly. The same trend was observed for IBP, which also showed a concentration-dependent decrease in fluorescence wavelength. As shown in Figure S1, *A* increase is the GFM with an increased peak intensity at 342 nm, and *A*′ increase is the GFM with an increased peak intensity at 350 nm. In the second component, *A* increase increased and *A*′ increase decreased with respect to the abscissa. This indicates that the larger the value of ω_2_ is, the shorter the peak wavelength is, and the smaller the value of ω_2_ is, the longer the peak wavelength is. Therefore, the second component was considered to reflect the behavior of the fluorescence wavelength. In the third component, unlike the first and second components, GFM in the model spectrum and KP, NPX, DZP, and IBP in the actual spectrum behaved quite differently. For example, KP and DZP monotonically attenuated BSA intrinsic fluorescence, but their behavior was not consistent with that of *A* decrease. IBP showed a concentration-dependent decrease in fluorescence intensity and a low wavelength shift, but its behavior was not consistent with neither *A* decrease nor *A*&*B* decrease nor *B* decrease in the third component. The large difference in behavior between the GFM generated by a single Gaussian function and the actual measurements suggests that the third component is a subtle change in the spectral pattern associated with drug binding to BSA.

In fact, NPX, the only one of the four drugs that increased its fluorescence intensity, behaved similarly to the other three drugs only in the third component. This is thought to indicate binding of NPX to BSA, which cannot be confirmed by looking at the fluorescence spectrum. Figure 3D summarizes the interpretation of each component shown so far. Figures S9 and S10 show the results of SVD processing of the data set without GFM. Compared to Figure S7A, Figure S9A reproduces the data set up to the third component to the same degree. However, the basis vectors of each component shown in Figure S9B indicate that the third component is mostly noise and has almost no effect on the original fluorescence spectrum. Compared to Figure S7B, the basis vector λ_1_ of the first component in Figure S8B is similar and the basis vector λ_2_ of the second component is only inverted. It can be seen that the basis vector λ_3_ of the third component in Figure S7B is important in the fine pattern changes in the fluorescence spectrum associated with drug binding to BSA. From Figure S9, it can be seen that the events indicated by the principal component vector ω_*i*_ of each component cannot be understood unless the GFM is cast as a probe for each component.

In the third component, the principal component vectors ω_3_ of each drug all exhibit a saturation curve-like behavior. If they were adsorption curves, the binding of NPX with increased fluorescence intensity, KP with decreased fluorescence intensity, DZP, and IBP to BSA could be evaluated uniformly. Therefore, we examined whether this saturation-like curve indicates drug binding to BSA, which is generally considered to be the Langmuir type.

### 3.3 Adsorption of Each Drug to BSA Revealed by SVD Including GFMs Regardless of Increasing-Quenching Fluorescence

**Figure 4:**
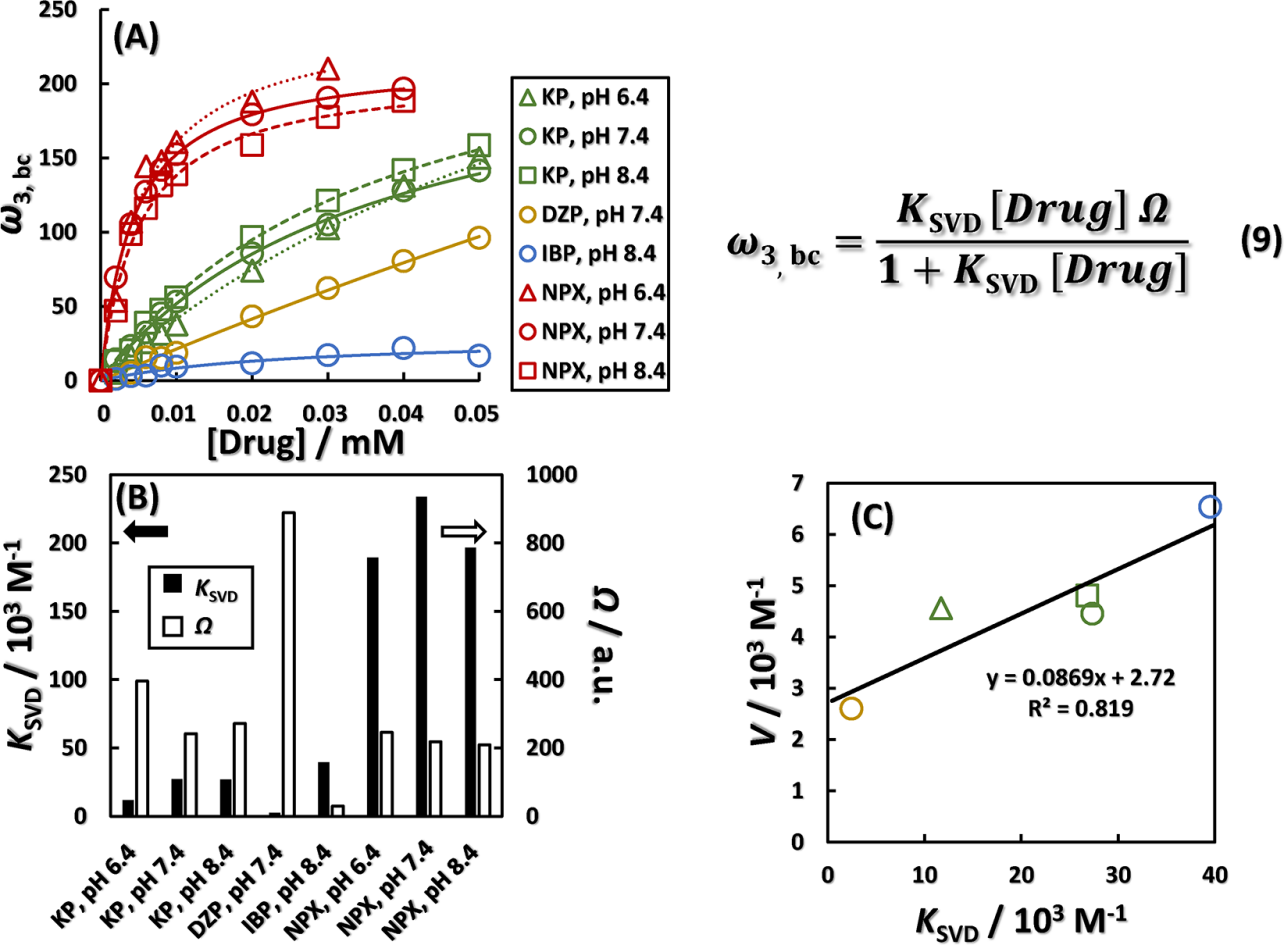
Application of Langmuir adsorption isotherms to saturation curves of baseline-corrected ω_3_ (ω_3,bc_) obtained by SVD. (A) Saturation curves of optimized eq 4 for ω_3,bc_ for each drug. (B) Binding constant *K*_SVD_ of each drug to BSA and saturation point of ω_3,bc_ calculated by eq 4. (C) A positive correlation between the static quenching constants *V* and *K*_SVD_, respectively. Green triangle: KP, pH 6.4, green circle: KP, pH 7.4, green square: KP, pH 8.4, yellow circle: DZP, pH 7.4, blue circle: IBP, pH 8.4.

We examined whether the saturation-like curve with the third component principal component vector ω_3_ identified in Figure 3C is indicative of drug adsorption on BSA. Figure 4A shows a baseline-corrected plot of the principal component vector ω_3_ of the third component for each drug in Figure 3C, with the starting point at the origin. The values were curve-fitted with the Langmuir-type adsorption (eq 9). Although Figure S11 shows the concentrations of KP up to the denser concentrations, we proceed with the inference based on Figure 4A because it shows a similar trend. Figure 4B shows the resulting association constant *K*_SVD_, Ω, indicating ω_3,bc_ at the saturation point. The correlation between the *K*_SVD_ obtained here and the static extinction constant *V*, which corresponds to the association constant obtained by the modified Stern–Volmer equation, is verified in Figure 4C. As a result, a high positive correlation between *K*_SVD_ and *V* was obtained. Therefore, *K*_SVD_ enables us to uniformly evaluate the binding of NPX, which enhances BSA intrinsic fluorescence, and the binding of the three drugs, which decrease it, to BSA. Similar *K*_SVD_ values for KP, IBP, and NPX were calculated by isothermal titration calorimetry (ITC) in addition to the fluorescence method.^31,32^ For DZP, a similar association constant to *K*_SVD_ has been calculated by drug competition experiments using the fluorescence method.^33^ These results support that the *K*_SVD_ corresponds to the aggregation constant.

In Figure 4B, IBP and NPX exceeded the *K*_SVD_ of KP, which has been reported in many cases of photosensitivity. However, unlike KP, IBP is dominated by static quenching, and the mode of binding and the effect of binding on the conformation of BSA may be different from those of KP. Therefore, the temperature factor of the X-ray crystallographic analysis of SA and SA–drug complexes was used to confirm this interpretation.

### 3.4 Correspondence between the Temperature Factor of the Crystal Structure of SA and the Quenching Mechanism

Modified Stern–Volmer equation and SVD analysis showed that DZP has a low affinity for BSA and that IBP and NPX have a higher affinity for BSA than KP, which has been frequently reported to cause photosensitivity. Here, we evaluated the perturbation of Trp213, the source of intrinsic fluorescence of BSA, at or near the binding region of each drug based on the temperature factor of X-ray crystallographic analysis.

**Scheme 1:**
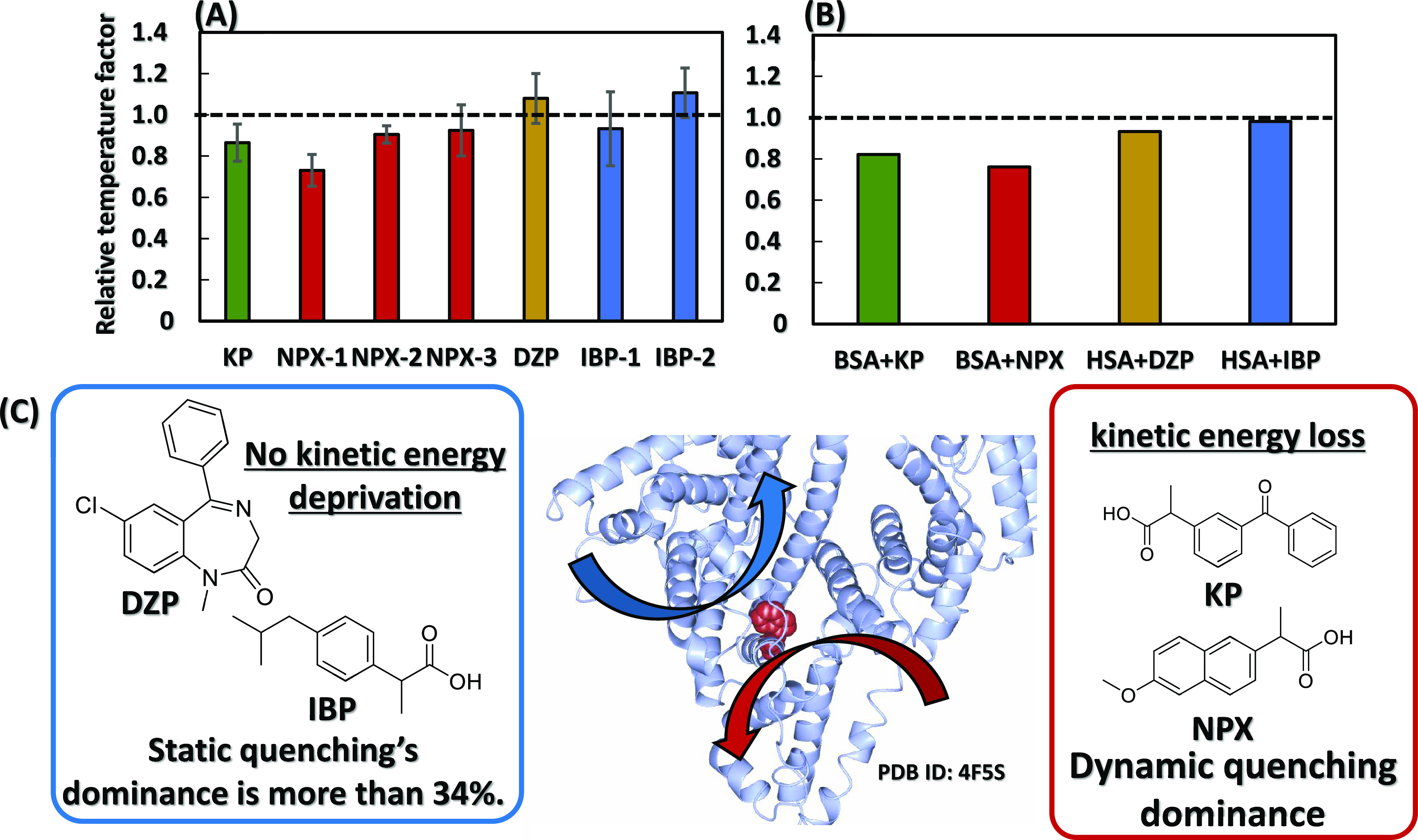
Variation in Temperature Factor of Protein Crystals Associated with Each Drug binding The vertical axis is the Relative temperature factor of (A) 6–14 amino acid residues adjacent to the binding drug/(B) Trp residues. Here, Relative temperature factor is the temperature factor of (A) average of amino acid residues or (B) Trp213/214, at drug binding divided by the temperature factor at drug non-binding. Temperature factor is calculated from crystallographic analysis of Scheme S3 and PDB IDs 4F5S and 1E78. (C) Overview on temperature factor and dynamic quenching

The temperature factor, B-factor, corresponds to the mean-square displacement of the crystal image of the kinetic properties of each amino acid residue in the plasma of the protein.^34^ For example, if a residue is involved in a covalent bond such as a disulfide bond, the temperature factor for that residue is lower. On the other hand, the temperature factor is higher for residues in loops that protrude from the globular mass of the protein.^34^ In the present study, the temperature factors of amino acid residues in the SA binding region of each drug or Trp residues were calculated based on the crystal structure data referred from the PDB shown in Scheme S3. Scheme 1A,B shows the results. The relative temperature factor on the vertical axis is the temperature factor of the complex of SA and each drug divided by the temperature factor of the corresponding amino acid residue in the crystal of SA alone. For DZP and IBP, for which no crystal data with BSA were available, the crystal structures of human SA (HSA) and its complex with HSA were used. The average relative temperature factor of the amino acid residues in the binding region of each drug is shown in Scheme 1A. The temperature factors of KP and NPX were slightly lower than those of SA alone, indicating that KP and NPX decreased the mobility of the binding region more than DZP and IBP. This indicates that the BSA binding region is strongly bound to KP and NPX. Since the intrinsic fluorescence analysis of proteins, including the modified Stern–Volmer equation, is based on microenvironmental changes around Trp residues, we also paid attention to the temperature factor of Trp residues. We calculated the relative temperature factor of Trp213 or Trp214, which are located near site II of SA and near the binding region of each drug, and the results are shown in Scheme 1B. DZP and IBP hardly changed the temperature factor for SA alone, while KP and NPX significantly decreased the temperature factor. This indicates that the kinetics of the Trp residues are decreased with the binding of KP and NPX.

KP, which was found to decrease the mobility around Trp213 by temperature factor analysis, was found to have a large dominant rate of dynamic quenching by analysis using the modified Stern–Volmer equation. In contrast, DZP and IBP, which also did not decrease the mobility around Trp residues, were found to have a high dominance of static quenching (>34%). This trend was attributed to the distance between the drug and Trp residues in each quenching mechanism. Static quenching occurs, as discussed for the static quenching constant *V*, even if the distance to the Trp residue is not as close as for dynamic quenching. Since dynamic quenching is quenching caused by intermolecular collisions, the drug must be closer to the Trp residues.^35^ Therefore, it was thought that dynamic quenching occurs in NPX as well as in KP but that it is not easily observed due to the fluorescence of NPX itself. Therefore, as shown in Scheme 1C, the binding mode of each drug to BSA site II can be divided into two. Taking into account that the homology between BSA and HSA is about 60%, it is difficult to make a clear argument on the degree of difference in Scheme 1A. However, the difference between KP/NPX and DZP/IBP in Scheme 1B is due not only to the difference in SA properties but also to the physical properties and basic skeleton of the drugs.

Finally, we discuss p*K*_a_ of each drug, although it is complicated. The p*I* of BSA is 4.7. Thus, at pH 6.4–8.4, BSA is present with an overall negative charge. If each drug is bound in a reaction centered on electrostatic interactions, DZP should bind more predominantly than the other drugs, but rather, the opposite is the case. Therefore, in this study, the change in affinity of each drug for BSA in response to changes in pH is not due to p*K*_a_, but to conformational changes in BSA, such as NB transition. This is why in the following sections, we focus on the molecular structure of the drugs rather than their electrostatic interactions. In addition, while there is a tendency to shy away from using BSA rather than HSA in drug-related studies, there are logical reasons for using BSA in this study. Here, we focus on site II where Trp213 is located in the vicinity, and Trp residues are also present in that region in HSA. However, when we extend our study to site I in the future, HSA without Trp residues around site I may provide less information than BSA. Indeed, we were able to evaluate the fluorescence behavior of the two Trps of BSA separately in a previous study.^17^ From this perspective, we believe it is reasonable to start with the BSA.

In the present study, the parameters of X-ray crystallography were used to support the classification of the binding mode of drugs to SA by ω_3_. This does not indicate that it cannot be used for proteins for which the crystal structure is not known. For the purpose of observing the complexes that arise in blood, crystal structures that show only the structure of a dried protein are not reliable. Furthermore, some combinations of protein–drug complexes are very difficult to crystallize and analyze. Therefore, the classification of drug binding mode by ω_3_ that we proposed in this study is important. The behavior of protein intrinsic fluorescence depends on the conformational change of the protein in solution and the binding of the drug. Therefore, ω_3_ obtained based on this fluorescence behavior is suitable for observing the binding mode in solution. Regardless of whether the crystal structure can be observed or not, the method can be extended to other drugs than those treated in this study. In this study, we found the characteristics of drugs that bind to SA from ω_3_ and cause photosensitivity. Furthermore, it is expected that the risk of photosensitivity of any drug can be determined by whether or not the behavior of ω_3_ belongs to the same group as that of KP and NPX.

## 4 Conclusions

In this study, we focus on SA site II to which KP is known to cause drug-induced photosensitivity, and classify the binding modes of site II drugs KP, DZP, IBP, and NPX to SA by fluorescence analysis. For KP, DZP, and IBP, where drug-concentration-dependent quenching of BSA-intrinsic fluorescence was observed, the modified Stern–Volmer plot showed that dynamic quenching was dominant for KP and static quenching was dominant for DZP and IBP. However, this analysis does not allow comparison of NPX with the three drugs. Therefore, by simultaneous SVD of the measured fluorescence spectrum and GFMs, a fine spectral pattern change accompanied by the binding of each drug in the spectrum to BSA was found as the behavior of principal component vector like saturation curve depending on drug concentration. The *K*_SVD_ obtained by the Langmuir equation for this saturation curve showed a high correlation with the static extinction constant *V*. Therefore, *K*_SVD_ indicates the association constant of the drug with BSA and it was found that NPX and IBP had higher values than KP. Finally, in the analysis of the temperature factor, KP and NPX showed a significant decrease in the temperature factor while DZP and IBP showed almost no change. This result is consistent with the dominance of dynamic quenching and static quenching in the quenching mechanism. In this study, SVD was used to investigate the interaction between SA and drugs, which has been widely studied in fluorescence quenching studies. SVD enabled us to extract information on drug adsorption to BSA from fluorescence spectra, which was consistent with the results of quenching studies and X-ray crystallography. Furthermore, this technique allowed us to evaluate the binding of NPX to BSA by fluorescence spectroscopy, where quenching studies could not be applied. The application of SVD is not limited to NSAIDs and cytomarkers but is expected to make it possible to perform fluorescence spectroscopy for drug binding to proteins without being limited by the fluorescence properties of the drug. The present results show that NPX has a higher affinity for BSA than KP and that NPX binds more strongly to BSA by the same mechanism as KP. KP, NPX, and IBP, all of which had large *K*_SVD_, all had propionate moieties. KP and NPX, which were considered to have a similar binding mechanism, have one more aromatic ring than IBP. The binding of KP and NPX to Trp residues, which are aromatic amino acids, is considered to be strong enough to cause dynamic quenching via π–π stacking interactions. DZP also has multiple aromatic rings, but the reason why the results are quite different from those of NPX and KP is that it does not have a propionic acid group. It was shown that penetration into the hydrophobic core inside BSA can be achieved not only by one of multiple aromatic rings and propionic acid groups but also by the joint effect of both. A further argument is that these bindings reduce the mobility of the SA hydrophobic core around Trp, which is thought to cause SA to become antigenic in photosensitivity.

## Supporting Information Available

Gaussian functional model (GFM) used to interpret each component obtained by decomposing the fluorescence spectra by SVD; drug-concentration-dependent quenching and increase of BSA intrinsic fluorescence; fluorescence spectrum of BSA in the presence of NPX-subtracted fluorescence of NPX alone; application of Stern–Volmer plots to drug concentration-dependent quenching of BSA intrinsic fluorescence and Stern–Volmer quenching constant (*K*_SV_) for KP, DZP, and IBP; application of modified Stern–Volmer plots to drug concentration-dependent quenching of BSA intrinsic fluorescence; each drug binds to BSA in a 1:1 stoichiometric ratio; singular values (σ_*i*_) and basis vectors (ψ_*i*_) obtained as a result of SVD processing for the fluorescence spectra and GFM; interpretation of each component by SVD including GFM; singular values (σ_*i*_) and basis vectors (ψ_*i*_) obtained as a result of SVD processing for the fluorescence spectra; interpretation of each component by SVD not including GFM; application of Langmuir adsorption isotherms to saturation curves of baseline corrected ω_3_ (ω_3,bc_) obtained by SVD; the outline of the SVD for fluorescence spectra and GFM; interpretation of each component by SVD including GFM; single-crystal X-ray structure data obtained from PDB illustrated in CCP4MG (PDF)

## Abbreviations

SA: serum albumin
BSA: bovine serum albumin
HSA: human serum albumin
KP: ketoprofen
DZP: diazepam
IBP: ibuprofen
NPX: naproxen
SVD: singular value decomposition
GFM: Gaussian function model spectrum
