# Supplementary material for: Propionic Acid Groups and Multiple Aromatic Rings Induce Binding of Ketoprofen and Naproxen to the Hydrophobic Core of Bovine Serum Albumin
Source: Mol Pharm. 2023 Jun 20;20(7):3549–58. doi: 10.1021/acs.molpharmaceut.3c00169 (PMC10324393; doi:10.1021/acs.molpharmaceut.3c00169)
Supplement: Supplementary file 1 — mp3c00169_si_001.pdf [file mp3c00169_si_001.pdf]

Propionic acid groups and multiple aromatic rings induce binding of ketoprofen and naproxen to the hydrophobic core of bovine serum albumin

Minori Tsurushima, Yuya Kurosawa, Satoru Goto

Supporting Information

$$I = A \exp\left\{\frac{-(x-B)^2}{2C^2}\right\}$$

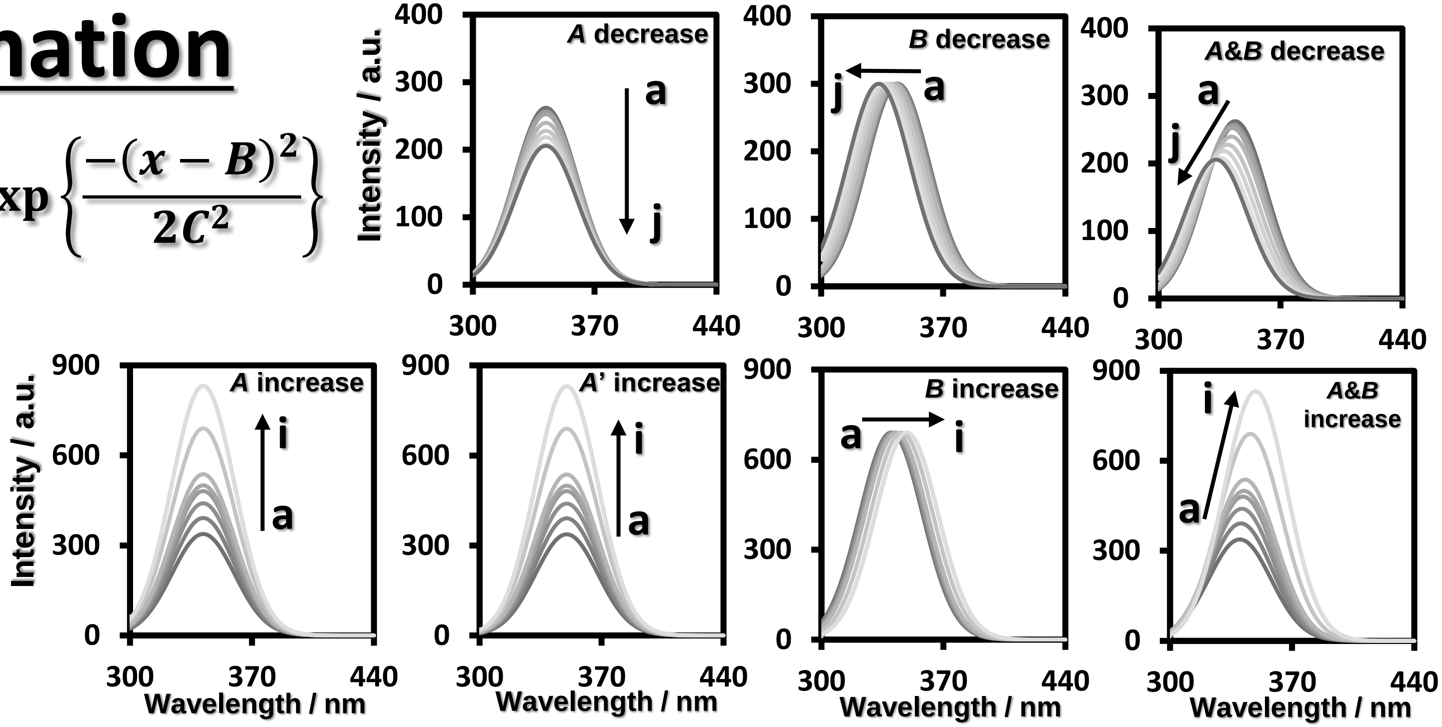

Fig. S1. Gaussian functional model (GFM) used to interpret each component obtained by decomposing the fluorescence spectra by SVD. These are single Gaussian functions varying only the indicated parameters with respect to drug concentration for convenience. Here, *A* corresponds to the fluorescence intensity and B corresponds to the fluorescence wavelength in the fluorescence spectrum. *B* = 342 when indicated as *A*, and *B* = 350 when indicated as *A*'. a-j: 0, 2, 4, 6, 8, 10, 20, 30, 40, 50 μM drug.

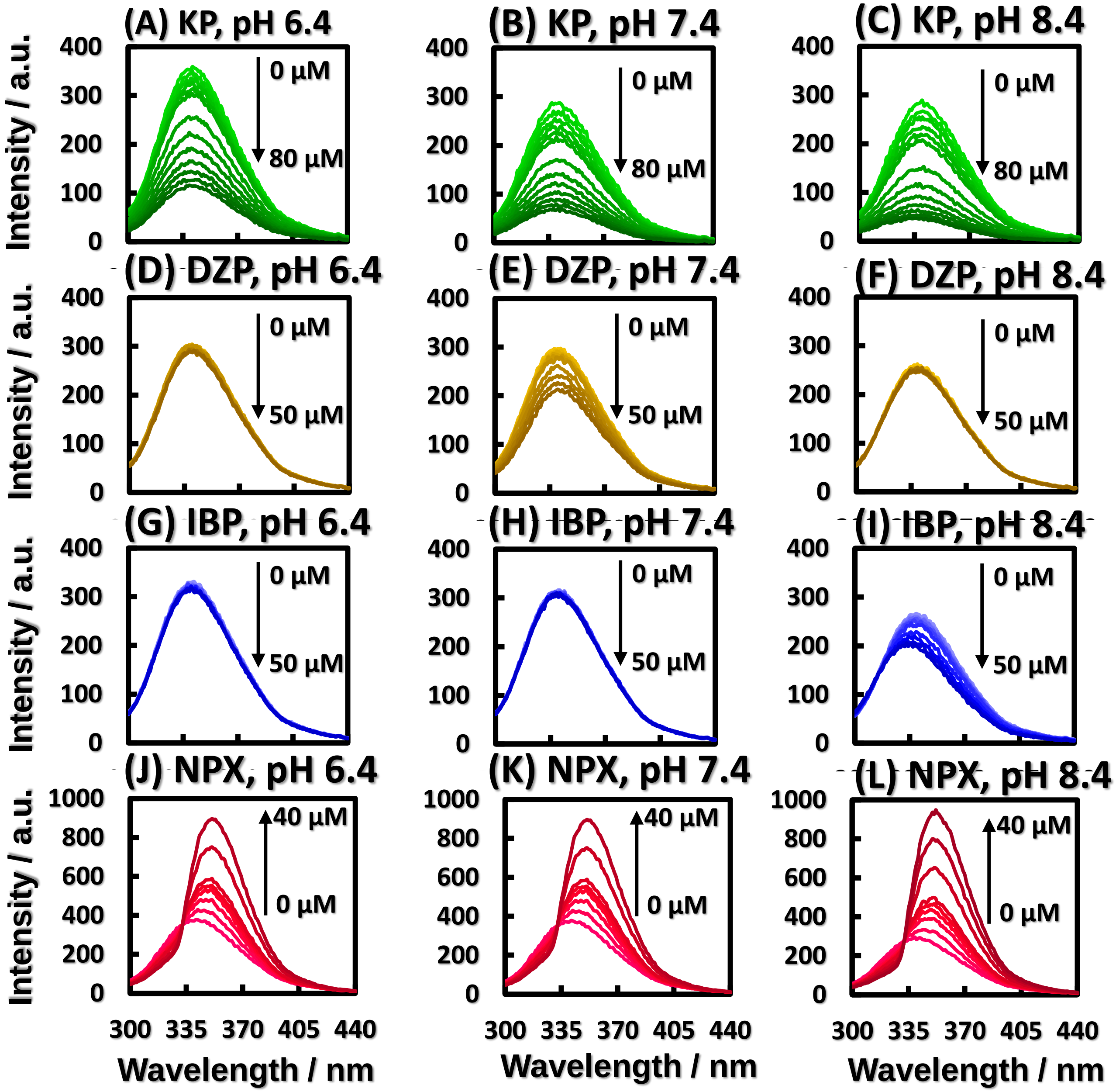

Fig. S2. Drug-concentration-dependent quenching and increase of BSA intrinsic fluorescence.
Fluorescence spectra of BSA with (A): KP at pH 6.4, (B): KP at pH 7.4, (C): KP at pH 8.4, (D): DZP at pH 6.4, (E): DZP at pH 7.4, (F): DZP at pH 8.4, (G): IBP at pH 6.4, (H) IBP at pH 7.4, (I): IBP at pH 8.4, (J): NPX at pH 6.4, (K) : NPX at pH 7.4, and (L): NPX at pH 8.4. [BSA] = 5.0 μM. Note that (B), (E), (I) and (K) are also shown in Fig. 1

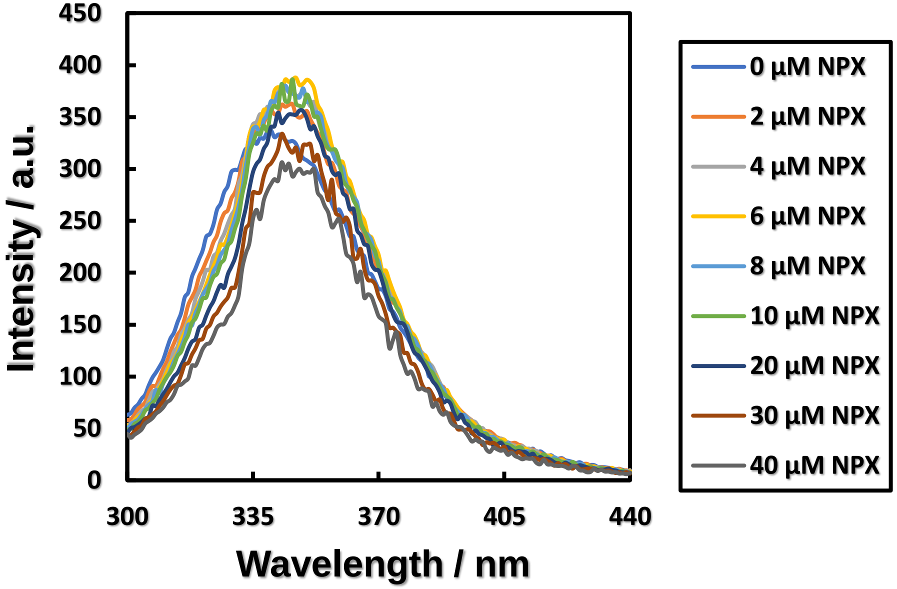

Fig. S3. Fluorescence spectrum of BSA in the presence of NPX subtracted fluorescence of NPX alone. In other words, Fig. 1E is subtracted from Fig. 1D.

$$\frac{F_0}{F} = 1 + K_{SV}\,[Drug] \qquad (1)$$

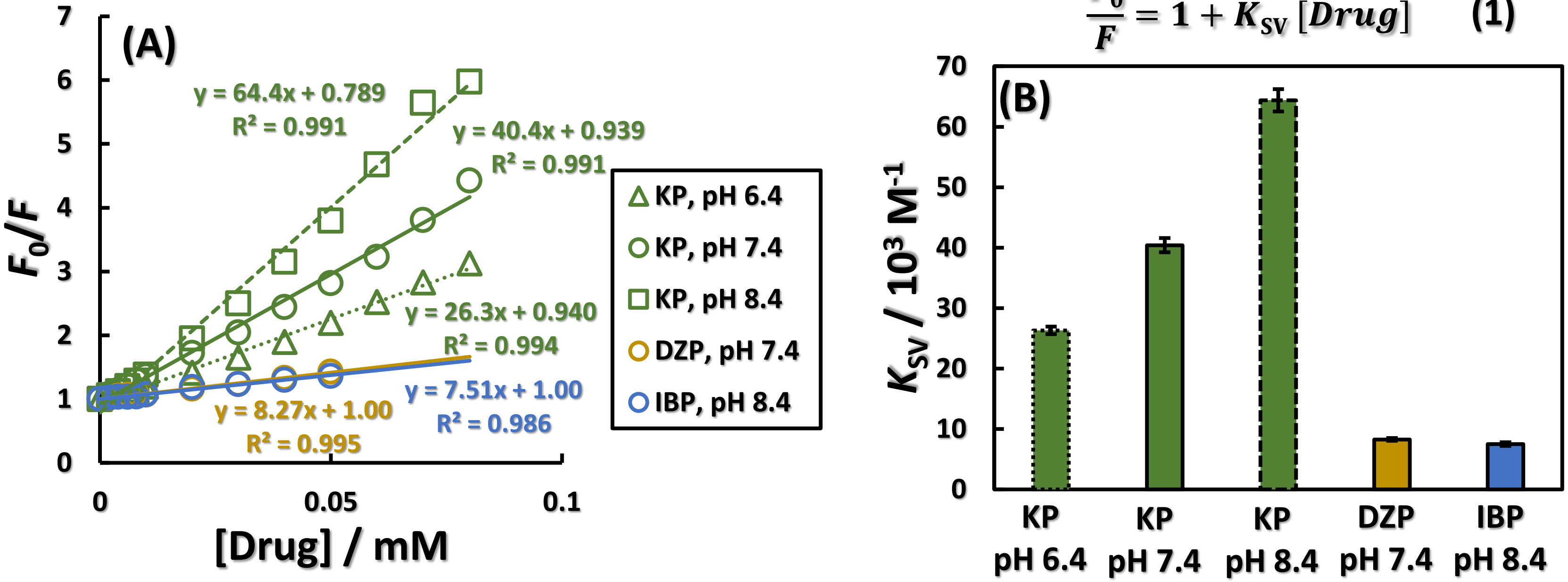

Fig. S4. Application of Stern Volmer plots (A) to drug concentration-dependent quenching of BSA intrinsic fluorescence. (B): Stern Volmer quenching constant (K_{SV}) for KP, DZP and IBP.

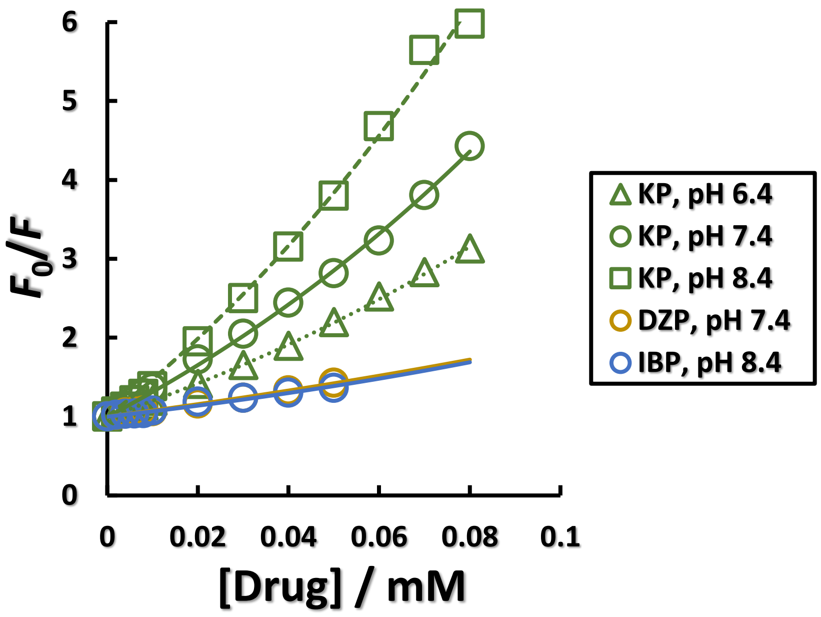

Fig. S5. Application of modified Stern Volmer plots to drug concentration-dependent quenching of BSA intrinsic fluorescence. This graph corresponds to Fig. 2(A) and illustrates KP up to higher concentrations (80 µM).

$$\ln \frac{F_0 - F}{F} = \log K_a + n \log[Drug] \quad (4)$$

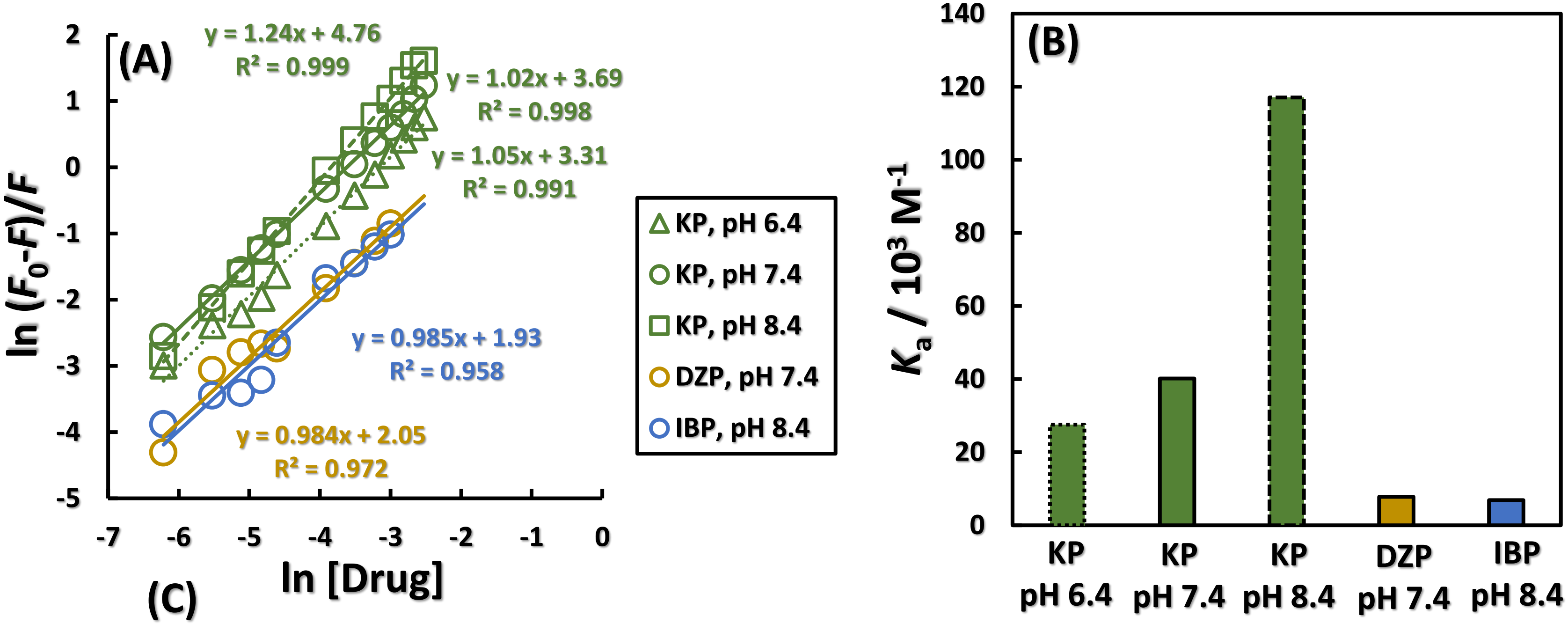

(C)

Drug	KP, pH 6.4	KP, pH 7.4	KP, pH 8.4	DZP, pH 7.4	IBP, pH 8.4
n	1.05	1.02	1.24	0.984	0.985

Fig. S6. Each drug binds to BSA in a 1:1 stoichiometric ratio. (A): The line shown by Eqn (1) optimized for the measured value. (B): Binding constant K_a of each drug to BSA calculated by Eqn (1). (C): Number of binding sites *n* for each drug to BSA calculated by Eqn (1)

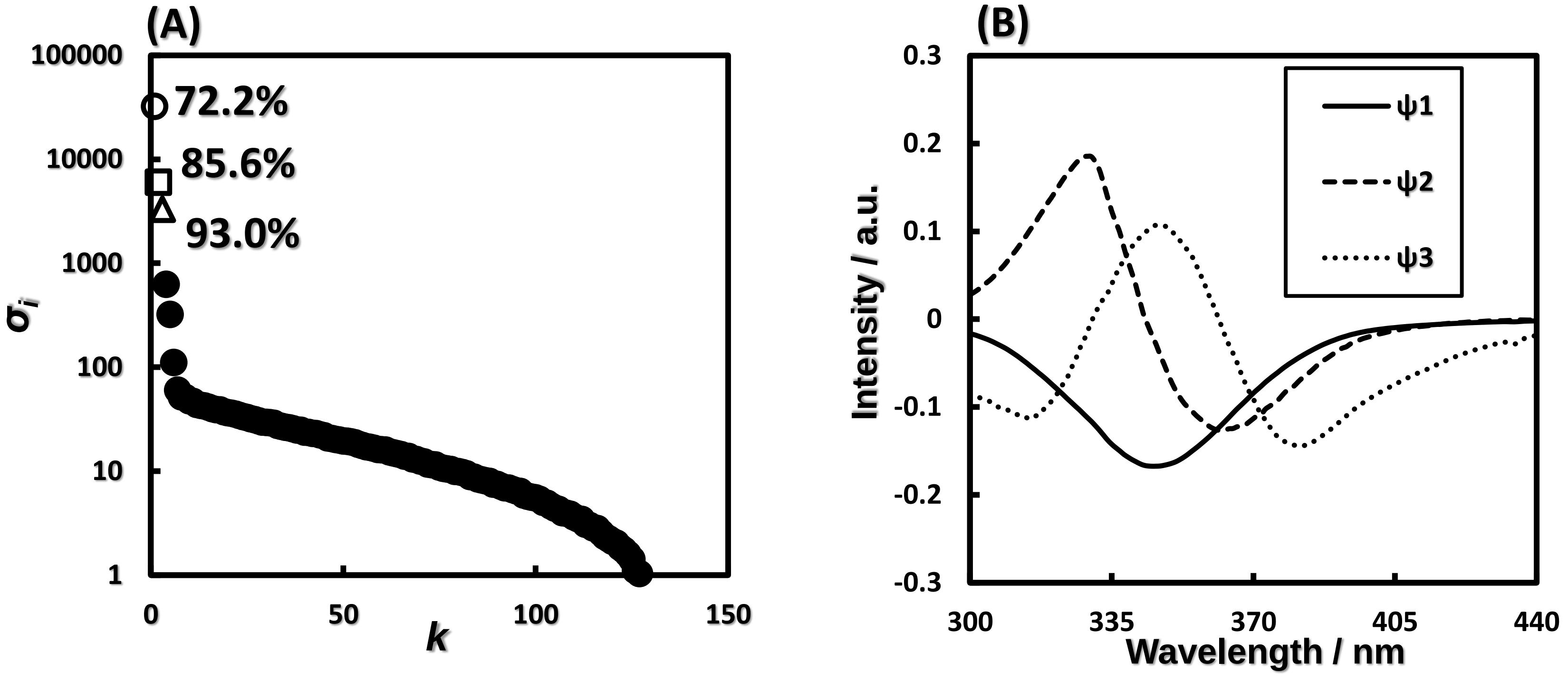

Fig. S7. (A): Singular values (σ_i) and (B): basis vectors (ψ_i) obtained as a result of SVD processing for the fluorescence spectra and GFM shown in Fig. 1., Fig. S1. and Fig. 7. σ_i indicates the contribution of each component to the original dataset. The dataset is reproduced by the contribution of ψ_i to the dataset and the increase or decrease of ψ_i. In (A), the values marked near the plots are the cumulative contribution to the dataset up to that component.

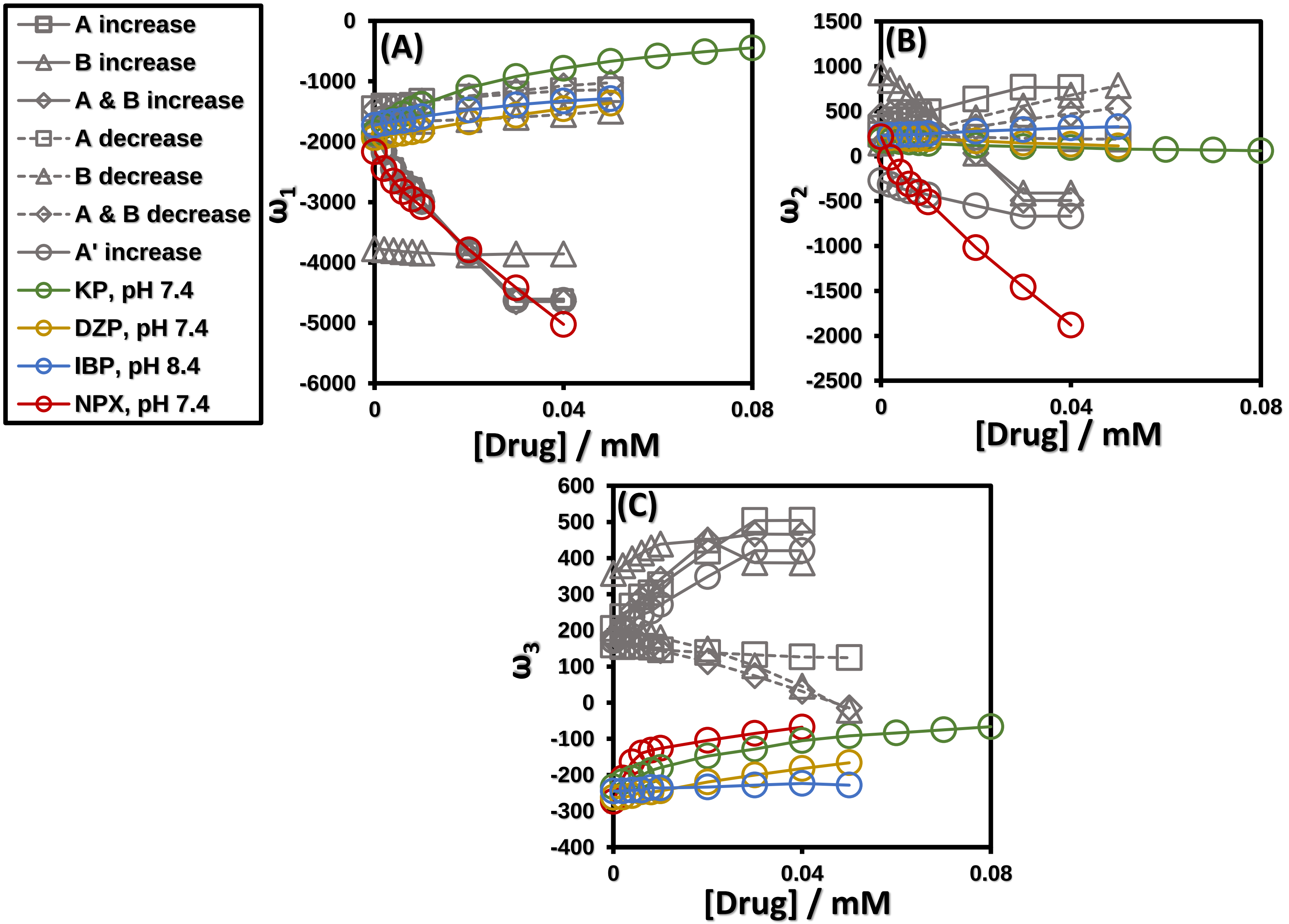

Fig. S8. Interpretation of each component by SVD including GFM. Variation of ω_1 (A), variation of ω_2 (B), and variation of ω_3 (C) with respect to drug concentration. Green, yellow, blue and red lines are experimental values. Gray solid and dashed lines are GFM value. These graph correspond to Fig. 3. and illustrate KP up to higher concentrations (80 μM).

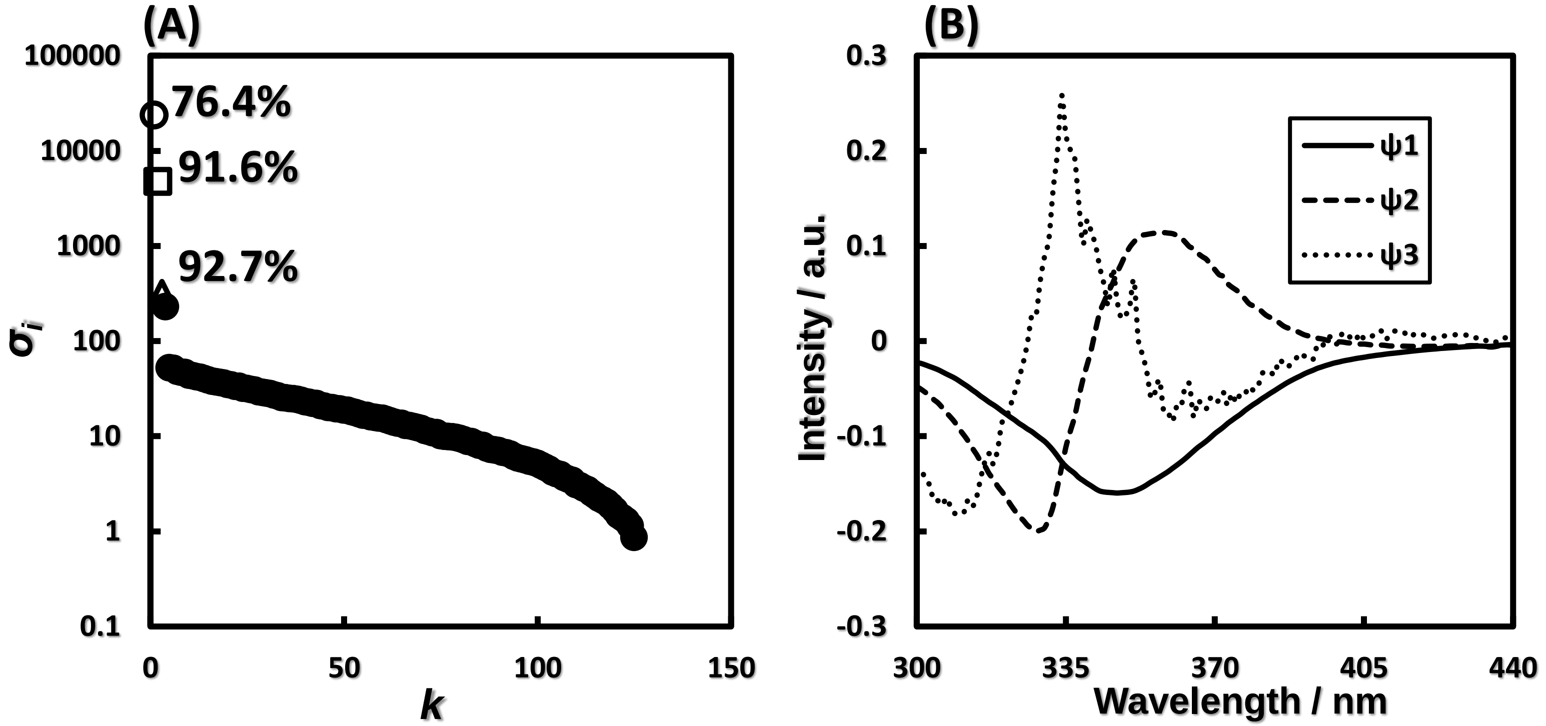

Fig. S9. (A): Singular values (σ_i) and (B): basis vectors (ψ_i) obtained as a result of SVD processing for the fluorescence spectra shown in Fig. 1. and Fig. S1. In (A), the values marked near the plots are the cumulative contribution to the dataset up to that component.

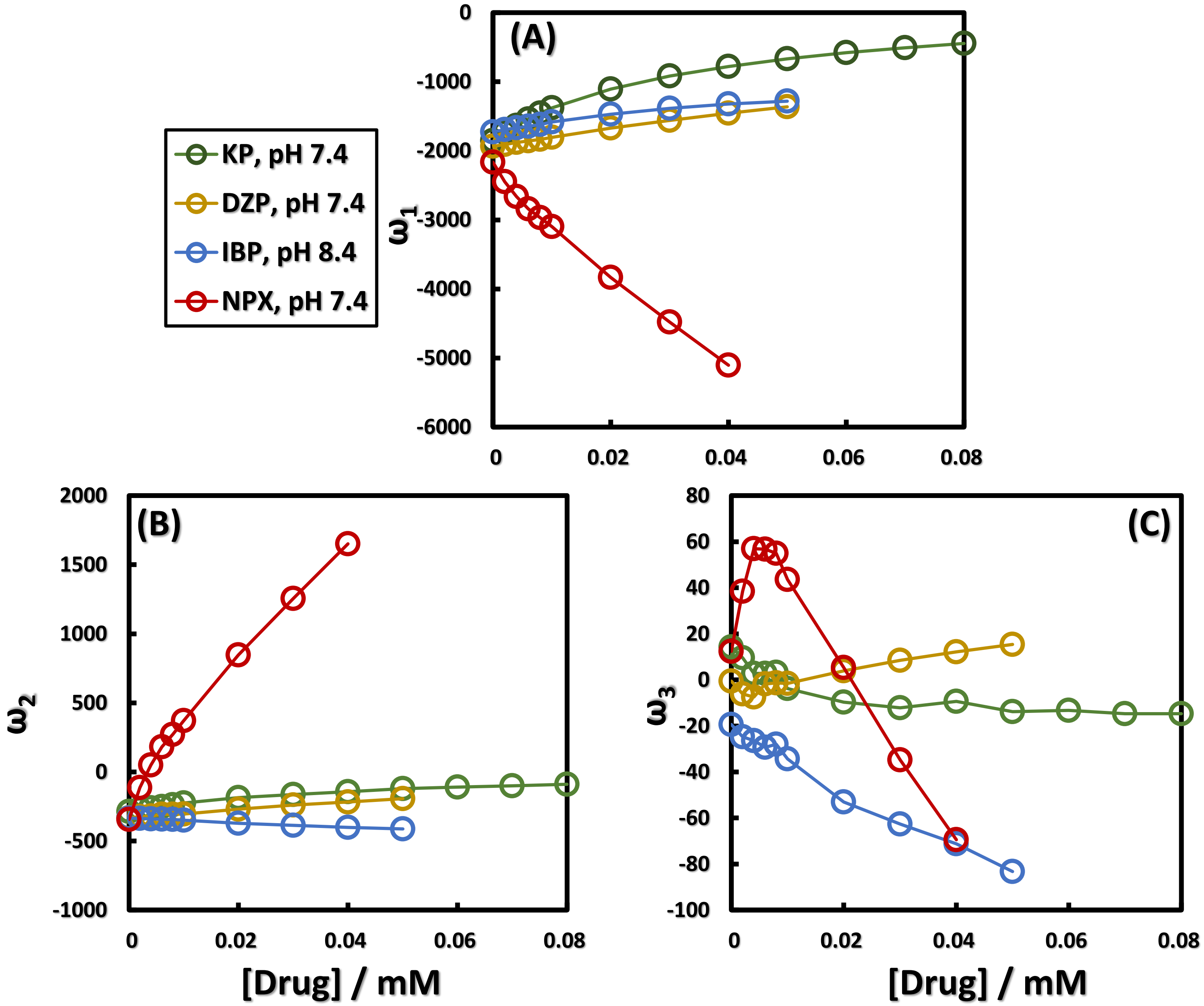

Fig. S10. Interpretation of each component by SVD not including GFM. Variation of ω_1 (A), variation of ω_2 (B), and variation of ω_3 (C) with respect to drug concentration.

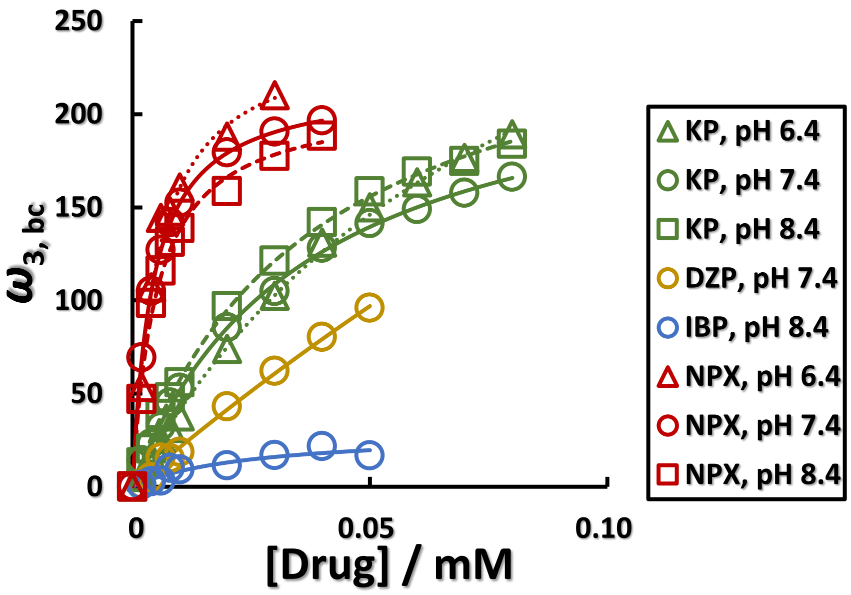

Fig. S11. Application of Langmuir adsorption isotherms to saturation curves of baseline corrected ω_3 ($\omega_{3,\ bc}$) obtained by SVD. Saturation curves are optimized Eqn (9) for $\omega_{3,\ bc}$ for each drug. This graph corresponds to Fig. 4A. and illustrates KP up to higher concentrations (80 µM).

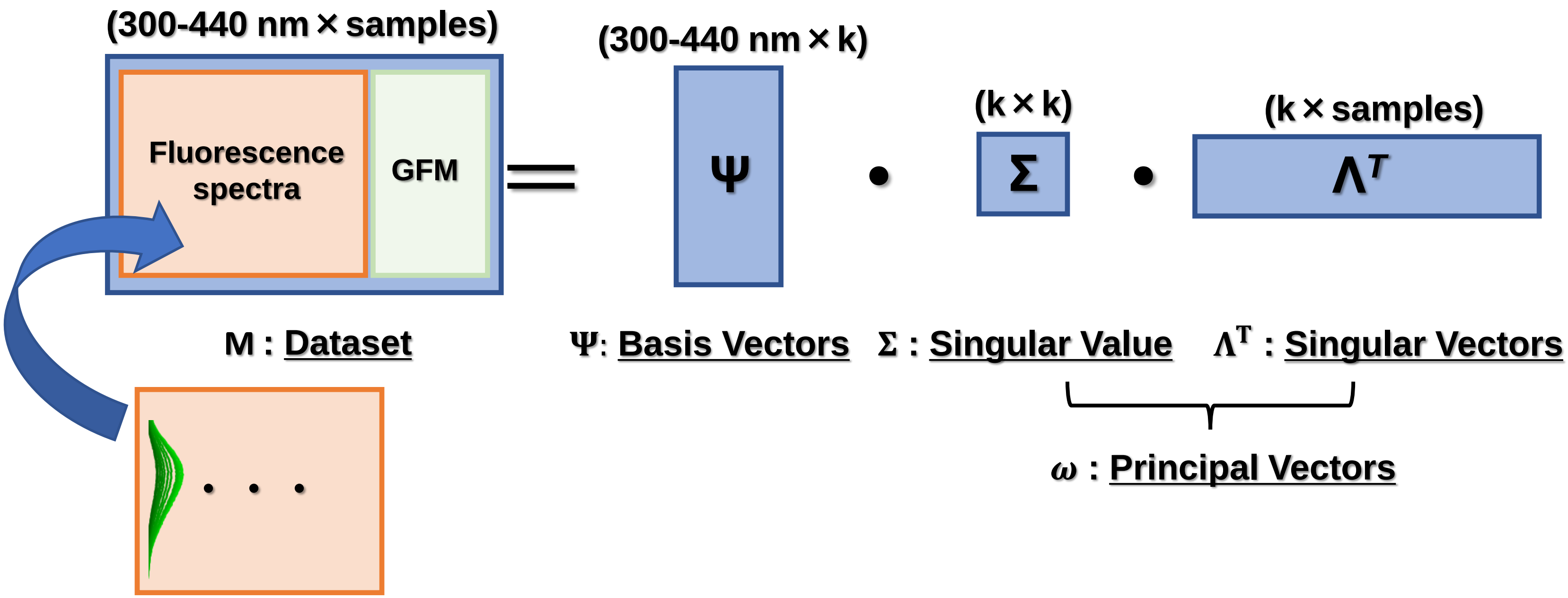

Scheme. S1. The outline of the SVD for fluorescence spectra and GFM. The dataset contains fluorescence spectra arranged so that the rows are the wavelengths, and the columns are the individual samples. The values in the matrix are the fluorescence intensities. The dataset (M) is decomposed into basis vectors (Ψ) that exhibit wavelength-dependent behavior and singular vectors (Λ) that exhibit sample-dependent behavior. Singular values (Σ) indicate the contribution to the dataset.

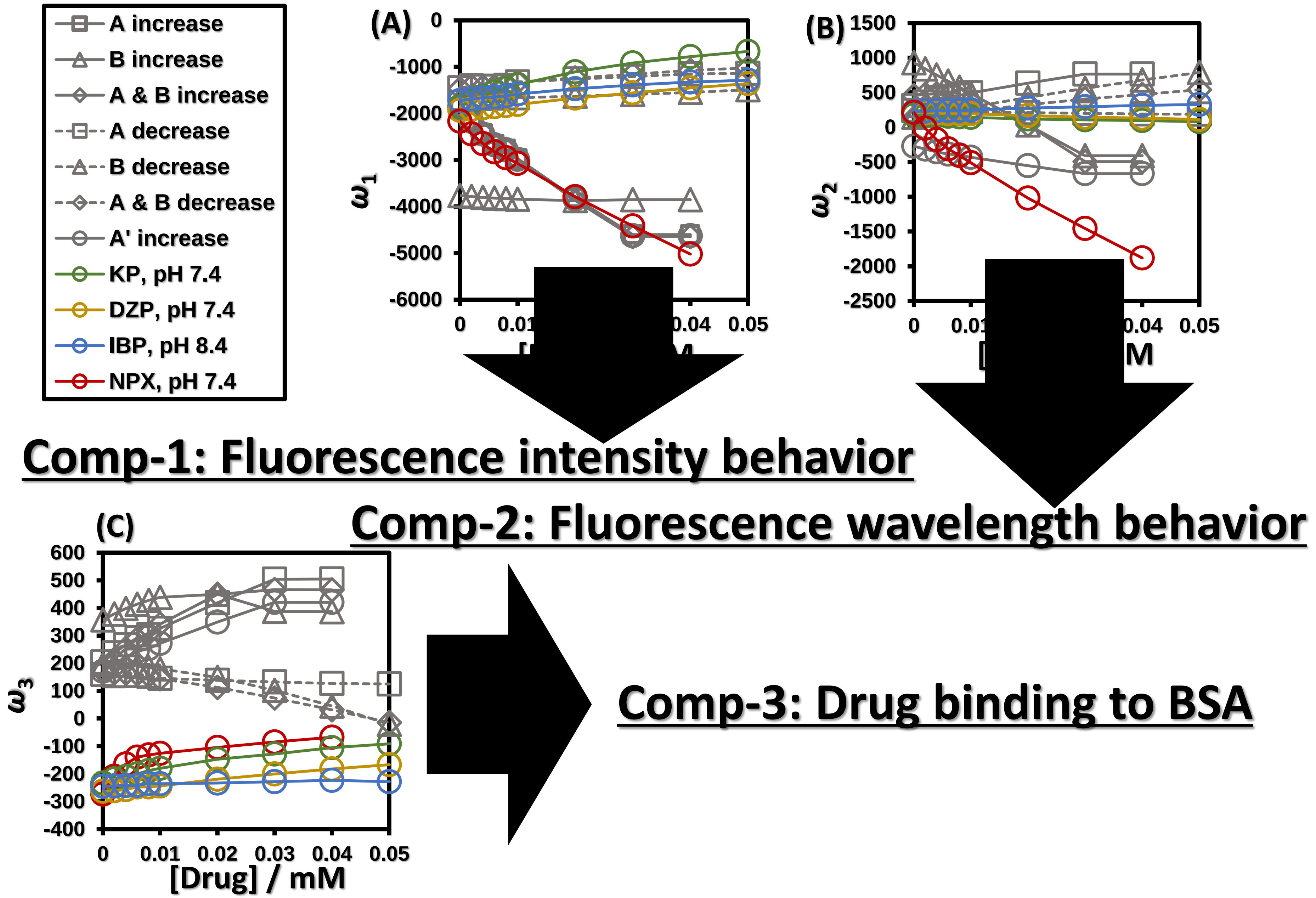

Scheme. S2. Interpretation of each component by SVD including GFM. Variation of ω_1 (A), variation of ω_2 (B), and variation of ω_3 (C) with respect to drug concentration. Green, yellow, blue and red lines are experimental values. Gray solid and dashed lines are GFM value. These graphs themselves are the same as Fig. 3.

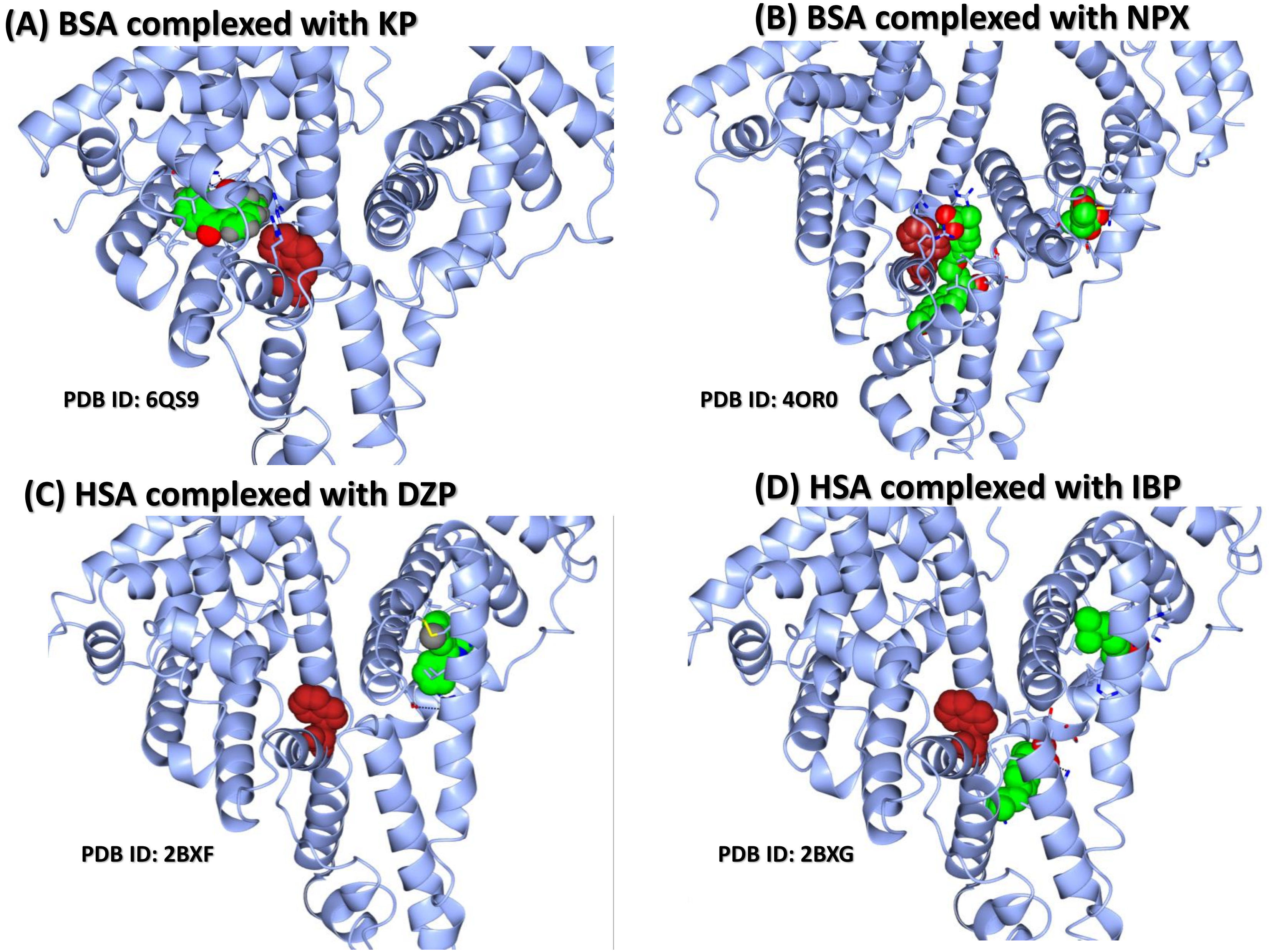

Scheme. S3. Single crystal X-ray structure data obtained from PDB are illustrated in CCP4MG. Trp residues are shown in dark red, and drugs bound to BSA /HSA are shown in green, gray, and bright red. (A): BSA complexed with KP, (B): with NPX, (C): HSA complexed with DZP, (D): HSA complexed with IBP.
